# Temporal dietary shift in jellyfish revealed by stable isotope analysis

**DOI:** 10.1007/s00227-016-2892-0

**Published:** 2016-04-22

**Authors:** Jamileh Javidpour, Ashlie N. Cipriano-Maack, Agnes Mittermayr, Jan Dierking

**Affiliations:** GEOMAR Helmholtz Centre for Ocean Research Kiel, Düsternbrooker Weg 20, 24105 Kiel, Germany; Biological, Earth and Environmental Science, University College Cork, Cooperage Building, Distillery Fields, North Mall, Cork, Ireland; Marine Biological Laboratory, 7 MBL Street, Woods Hole, MA 02543 USA

## Abstract

A temporal change in the stable isotope (SI) composition of jellyfish in the Kiel Fjord, Western Baltic Sea, was documented by analyzing δ^13^C, δ^15^N and δ^34^S of bell tissue of *Aurelia aurita* and *Cyanea capillata* in the period between June and October 2011. A strong and significant temporal change in all SI values of *A. aurita* was found, including an increase of ~3 ‰ in δ^13^C, a decrease of ~4 ‰ in δ^15^N and sharp decline of ~7 ‰ in δ^34^S. While knowledge gaps in jellyfish isotope ecology, in particular the lack of reliable trophic enrichment factors, call for a conservative interpretation of our data, observed changes in particular in δ^34^S, as indicated by means of a MixSIR mixing model, would be consistent with a temporal dietary shift in *A. aurita* from mesozooplankton (>150 µm) to microplankton and small re-suspended particles (0.8–20 µm) from the benthos. Presence of a hitherto unidentified food source not included in the model could also contribute to the shift. During the 2-month occurrence of *C. capillata*, its isotope composition remained stable and was consistent with a mainly mesozooplanktonic diet. Mixing model output, mainly driven by δ^34^S values, indicated a lower proportion of *A. aurita* in the diet of *C. capillata* than previously reported, and thus to a potentially lesser importance of intraguild predation among jellyfish in the Kiel Fjord. Overall, our results clearly highlighted the potential for substantial intraspecific isotopic seasonal variation in jellyfish, which should be taken into account in future feeding ecology studies on this group.

**Electronic supplementary material:**

The online version of this article (doi:10.1007/s00227-016-2892-0) contains supplementary material, which is available to authorized users.

## Introduction

Global awareness has been drawn to the increase in jellyfish blooms due to their possible negative impacts on ecosystem goods and services, such as interference with tourism, aquaculture, fishing operations and coastal industrial intakes (Richardson et al. 2009; Condon et al. 2012). Population outbreaks of carnivorous jellyfish account for severe impacts on marine food webs, driven by a rapid population growth rate in combination with a highly successful competition for food sources (Hay 2006; Gibbons and Richardson 2013). Populations of *Aurelia aurita* medusae have been known to consume roughly two-thirds of daily secondary production (mainly copepods) and thus compete with fish larvae for resources in the Kiel Bight, Baltic Sea (Behrends and Schneider 1995; Schneider 1989). In order to determine the ecological role and impact of jellyfish on marine food webs, it is important to gain a thorough understanding of their trophic ecology by comprehending both the formation and structure of their blooms, as well as their likely role in the transfer of carbon and energy in the marine food web (Pitt et al. 2009).

In recent years, there has been a rapid rise in the use of stable isotope (hereafter SI) analysis as a tool for studying trophic ecology, which led to a better understanding of origin, pathways and fate of organic matter (Robinson 2001; Michener and Kaufman 2007). By comparing SI values of a consumer over time, information on trophic transfer, carbon and energy flux, and contribution of food sources to the diet of an organism can be gained (Kling et al. 1992; Cabana and Rasmussen 1996; Ponsard and Arditi 2000). δ^13^C and δ^15^N have been most commonly used to address ecological questions (review by Grey 2006), since carbon (C) isotopes are well suited to identify the primary carbon sources at the base of a food web (Peterson 1999) and nitrogen (N) isotopes are a good tracer of the trophic position of an organism (Cabana and Rasmussen 1996). The use of additional elements has increased recently, e.g., sulfur (S) isotopes can reveal whether a food web is driven by benthic or pelagic primary production (Hansen et al. 2009; Jaschinski et al. 2008).

For many groups of animals, information on temporal SI changes is already available (Carlier et al. 2007); however, despite their ecological importance, to date, this information is lacking for most species of jellyfish, leading to misinterpretation of trophic ecology of gelatinous taxa (Fleming et al. 2015; Pauly et al. 2009). At the same time, recent work by Fleming et al. (2015) highlights that such variation in jellyfish can be substantial. Here, we were interested in the strength and patterns in intraspecific seasonal variation in SI values of δ^13^C, δ^15^N and δ^34^S of the pelagic jellyfish species *A. aurita* and *C. capillata* during their bloom period (June–October 2011) in Kiel Fjord, western Baltic Sea. Secondly, we interpreted these values in the context of isotope composition of dietary sources to assess potential temporal changes in diet composition of these two species.

## Materials and methods

### Study location

**Fig. 1:**
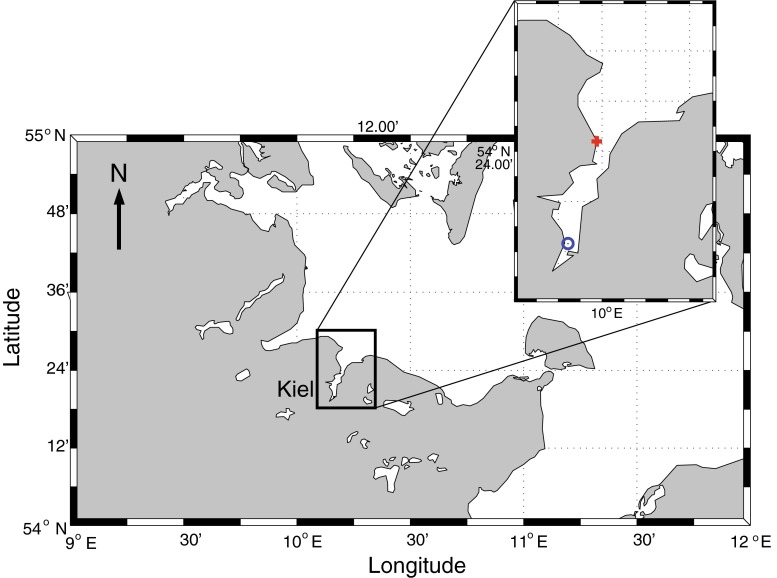
Study area in the Western Baltic Sea and the Kiel Fjord with sampling stations Witlingskuhle (*circle*) and Falkenstein station of Mittermayr et al. (2014b) (*plus sign*)

Kiel Fjord constitutes a small and shallow extension of the Kiel Bight in the Belt Sea (Fig. 1) with a mean depth of about 13 m (Javidpour et al. 2009). During most of the investigation period, the water column was well-mixed except during a short period of <15 days from July to August, where a weakly thermal stratification was detected.

### Sampling

Weekly sampling in the Kiel Fjord was carried out during the annual occurrence of jellyfish from June to November 2011. During this period, *A. aurita* occurred from June to the beginning of October and *C capillata* from the beginning of October to the end of November. A WP3 net with 1-mm mesh size was used to capture jellyfish by means of integrated vertical sampling between depths of 0 and 15 m. At each sampling event, five individuals per species were chosen from the collected material and bell diameter (inter-rhopalia) was recorded. Specimens were kept in filtered sea water for 2 h at 20 °C, after which no remaining prey items were observed in the guts, indicating that this period was sufficient to ensure complete gut evacuation (FitzGeorge-Balfour et al. 2013). Total wet mass of each individual was then measured to the nearest 0.01 g.

Prior to preparation for stable isotope analysis, the specimens were washed with filtered seawater (0.2 µm filter). Bell tissue of each individual, the most suitable body part for SI measurements in *A. aurita* (D’Ambra et al. 2014), was dissected, rinsed using milli-Q water, dried to constant dry weight at 50–60 °C and ground to a fine powder using mortar and pestle. Subsamples of 4 ± 0.05 mg, found to yield optimum results in initial analyses, were then weighed out and sealed in tin cups.

Stable isotope data of potential food sources for the same time period including seston and mesozooplankton were obtained from Mittermayr et al. (2014a). Seston samples were sieved through a 20-µm mesh to separate zooplankton and were then filtered on 0.8-µm cellulose acetate filters (Sartorius) and carefully scraped off into distilled water with plastic cell scrapers before being desiccated in small watch glasses. Since phytoplankton cannot be reliably separated from similar sized heterotrophic or detrital POM for stable isotope analysis, seston samples were treated as proxy for mixed microplankton food sources. A study by Sommer and Sommer (2004) supports this procedure as they were not able to find a clear connection between seston size fractions and their SI values. In the inner Kiel Fjord, seston can represent a mixture of phytoplankton and protozoans as well as re-suspended particles from benthos. Mesozooplankton samples were collected using a 150-μm mesh size plankton net. As spatial variation within the south and central Baltic Sea area only accounts for 0.4 % of the total variance in mesozooplankton isotopic values (Agurto 2007), the use of Mittermayr et al. (2014a) data were deemed plausible for comparative purposes in this investigation considering that sampling sites are only ~7 km apart.

### Stable isotope analysis

Analysis of samples was conducted with a continuous-flow isotope-ratio mass spectrometer (Europa Scientific ANCA-NT 20-20 Stable isotope analyzer with ANCA-NT Solid/Liquid Preparation Module) at the University of California at Davis’ stable isotope facility. Delta notation was used as follows:

$$\delta X\,(\permil) = [({R_{{\rm{sample}}}}/{R_{{\rm{standard}}}}) - 1] \times 1000$$

where *X* = ^15^N, ^13^C or ^34^S and *R* = ^15^N/^14^N, ^13^C/^12^C or ^34^S/^32^S. Reference materials for the calculation of δ-values were atmospheric N_2_ for N, Vienna Pee Dee Belemnite for C and SO_2_ for S. During analysis, samples were interspersed with replicates of two internal laboratory standards, nylon and bovine liver, previously calibrated against International Atomic Agency reference materials (IAEA-N1, IAEA-N2, IAEA-S-1, IAEA-S-2, IAEA-S-3 and USGS-40), in order to correct for drift. The long-term standard deviation was 0.2 ‰ for δ^13^C and δ^34^S, 0.3 ‰ for δ^15^N and 0.4 ‰ for δ^34^S.

Lipid content might severely affect δ^13^C values, resulting in ^13^C depleted values in correspondence with high lipid content and is therefore an important issue to address (DeNiro and Epstein 1977; Post et al. 2007). Post et al. (2007) advises to conduct lipid correction on δ^13^C values for aquatic animals if lipid content is higher than 5 % of the biomass, or if C:N ratios are higher than 3.5. Since this was the case for C:N ratios of both *A. aurita* and *C. capillata* (see Table 1), δ^13^C values were corrected for lipid content based on the methods of Post et al. (2007) and D’Ambra et al. (2014). Both methods led to relatively small shifts in δ^13^C and very similar patterns over time compared to our original values. However, while the Post et al. correction slightly decreased variability in our dataset, the D’Ambra et al. correction introduced additional noise into the data set and increased the variability especially at the beginning of the season (supplementary Fig. S.1). Therefore, we decided to apply the correction by Post et al. to our original δ^13^C data set.

**Table 1:** Temporal biometric data (mean ± SD) collected for *A. aurita* and *C. capillata* from June to October 2011

Month (2011)	Species	Sample size (*n*)	Wet mass (g) (±SD)	Length (cm) (±SD)	C (µg 4 mg DW^−1^) (±SD)	N (µg 4 mg DW^−1^) (±SD)	C:N Molar ratio
June	*A. aurita*	9	204.2 (92.1)	16.5 (3.6)	26.1 (9.1)	16.5 (3.6)	5.4 (0.9)
July	*A. aurita*	15	530.9 (355.3)	22.8 (6.9)	25.0 (18.7)	6.5 (5.2)	4.7 (0.9)
August	*A. aurita*	20	367.5 (256.3)	19.8 (5.3)	72.7 (42.6)	18.9 (10.9)	4.5 (0.1)
September	*A. aurita*	10	161.19 (95.0)	17.0 (3.2)	28.73 (13.4)	7.23 (3.6)	4.7 (0.67)
September	C. capillata	11	157.6 (97.5)	12.4 (3)	73.0 (27.4)	19.4 (6.6)	3.7 (0.4)
October	C. capillata	10	232 (161)	13.6 (4)	69.9 (25.3)	19.3 (7)	3.6 (0.2)

### Calculation of dietary composition based on MixSIR

To determine potential contributions of different food sources to the diet of the collected jellyfish, a mixing model (MixSIR) based on Bayesian probability was applied. MixSIR is a graphical user interface (GUI) built on MATLAB that employs an algorithm based on a Bayesian framework to determine the probability distributions for proportional contributions of each food source to the diet mix of a consumer (Semmens and Moore 2008). This model allows for allocation of different fractionation factors ± standard deviation (SD) for each element and source, respectively, and accounts for uncertainty in isotope values when estimating contributions of sources.

Fractionation values of 0.5 ± 0.5 ‰ for δ^13^C (France and Peters 1997; Jaschinski et al. 2011) and 0 ± 0.2 ‰ for δ^34^S (Michener and Kaufman 2007) were chosen for all trophic level transfers; for δ^15^N, 2.4 ± 1.1 ‰ and 3.4 ± 1.1 ‰ were chosen for the first and following trophic level transfers, respectively (Currin et al. 1995; Vanderklift and Ponsard 2003; Zanden and Rasmussen 2001). MixSIR was run with δ^13^C, δ^15^N and δ^34^S values of *A. aurita* and *C. capillata* on a bi-weekly basis. To account for turnover rates as reported by D’Ambra et al. (2014), where bell tissue of *A. aurita* reached SI steady state with laboratory diet after 18–20 days, a lag time of 2 weeks between stable isotope values of jellyfish and stable isotope values of their potential food sources was used in the model.

### Statistical analysis

Our initial data exploration for *A. aurita*, zooplankton and seston was carried out with the response variables δ^13^C, δ^15^N and δ^34^S and time (date) as explanatory variable following the protocol described in Zuur et al. (2010). The nonlinear relationship between response (SI) and explanatory variables (time) warranted the application of a generalized additive model (GAM) to δ^13^C, δ^15^N and δ^34^S. Data on *C. capillata* were analyzed by applying one-way ANOVA for each stable isotope value to determine differences among sampling time points as well as for comparison of SI values of *A. aurita* and *C. capillata.* All statistical assumptions such as normality and constant variances were checked for any analysis and were checked for outliers. Statistical analyses were performed in the software R 3.0.3 (R Core Team 2014).

Data management—raw data of the stable isotopes of jellyfish species underlying this paper are available at PANGAEA (http://doi.pangaea.de/10.1594/PANGAEA.858057).

## Results

### Seasonal changes in jellyfish occurrence, size and C:N ratios

*A. aurita* was present in all samples from June to September, whereas *C. capillata* was found only on four occasions in September and October. Biometric measurements of *A. aurita* indicated a significant increase in mean (±SD) diameter over time (*F*_(3,50)_ = 3.8, *p* = 0.01) with a steep increase from June (16.5 ± 3.6 cm) to July (22.8 ± 6.9 cm), followed by a decrease in individual mean size in August and September (19.8 ± 5.3 and 17.0 ± 3.1 cm, respectively). Maximum mean (±SD) wet mass was recorded in July (531 ± 355 g ind^−1^). Total carbon (µg) and nitrogen (µg) per 4 mg dry mass showed a peak in August, with 72.7 ± 42.7 and 18.9 ± 10.9 µg, respectively (mean ±SD). On average C:N ratios decreased from spring to summer and stayed constant until fall. Maximum C:N values were observed in June (5.4 ± 0.9), whereas the ratio was significantly lower in September (4.7 ± 0.7) (GAM, *F* = 59.1, *p* < 0.01).

In contrast, during the period of its occurrence (September–October), *C. capillata* showed neither evidence of growth nor change in wet mass or total carbon and nitrogen values (Table 1). C:N ratios also remained constant (3.7 ± 0.4 in Sep. and 3.6 ± 0.2 in Oct.).

### Temporal variability in jellyfish stable isotope values

**Fig. 2:**
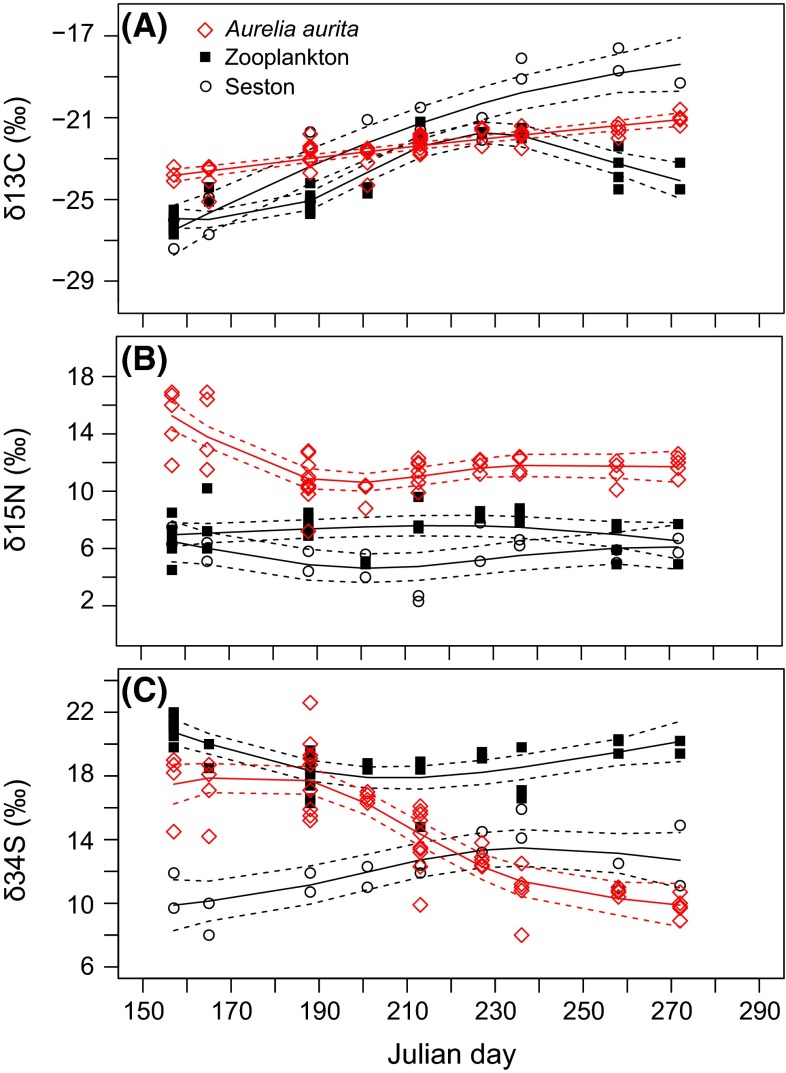
δ^13^C, δ^15^N and δ^34^S of *A. aurita* (*red diamond*), zooplankton (*square*) and seston (*circle*) over the course of 5 months in the Kiel Fjord (Jun–Oct 2011). Julian day 150 corresponds to May 30 and 290 to October 17

Strong directional temporal changes in all three isotopic markers occurred in *A. aurita* (Fig. 2a; Table 2). *A. aurita* δ^13^C values ranged from −23.9 ± 0.6 ‰ (mean ± SD) in June, to −21.3 ± 0.4 ‰ in September with a significant linear increase (GAM, *F* = 68.6, *p* < 0.001) toward the end of the season. While seston δ^13^C values were increasing significantly (GAM, *F* = 39.7, *p* < 0.001) from June (−26.0 ± 1.4) to September (−18.7 ± 1.1), zooplankton δ^13^C values increased from June (−25.2 ± 2.6) to the beginning of August (−21.8 ± 0.2), before decreasing from mid-August onward (GAM, edf = 3.7, *F* = 41.4, *p* < 0.001, Fig. 2a).

**Table 2:** Isotope values (mean ± SD) of potential food sources (after Mittermayr et al. 2014b) and jellyfish from June to October 2011

	June_1	June_2	July_1	July_2	August_1	August_2	September_1	September_2	October_1	October_2
*Seston*										
δ^15^N	6.3 (1.3)	5.1 ± 1.2	4.4 ± 1.4	4.0 ± 1.0	2.3 ± 0.2	5.1 ± 1.0	6.0 ± 0.9	5.0 ± 1.0		
δ^13^C	−26.0 ± 1.4	−25.0 ± 1.7	−21.6 ± 3.5	−21.1 ± 1.4	−20.5 ± 1.4	−21.0 ± 1.2	−19.1 ± 2.2	−18.7 ± 1.1		
δ^34^S	12.0 ± 3.2	9.9 ± 3.0	11.9 ± 1.2	11.9 ± 2.9	12.4 ± 0.2	13.2 ± 1.2	14.0 ± 2.9	12.4 ± 1.5		
*Zooplankton*										
δ^15^N	6.6 ± 2.0	7.7 ± 1.2	7.1 ± 1.1	6.1 ± 1.1	7.4 ± 0.6	7.3 ± 1.6	6.9 ± 1.9	6.5 ± 1.3		
δ^13^C	−25.2 ± 2.6	−24.6 ± 2.3	−25.1 ± 0.5	−23.1 ± 1.2	−21.8 ± 0.2	−22.1 ± 1.4	−23.0 ± 1.6	−21.8 ± 3.1		
δ^34^S	21.0 ± 1.0	18.2 ± 2.0	18.2 ± 1.1	18.6 ± 0.4	18.6 ± 1.2	18.9 ± 1.2	18.94 ± 1.4	19.1 ± 2.4		
*Aurelia*										
δ^15^N	15.1 ± 2.2	14.4 ± 2.6	11.0 ± 2.3	11.5 ± 2.2	11.2 ± 0.7	11.9 ± 0.5	11.3 ± 0.8	11.8 ± 0.7		
δ^13^C	−22.0 ± 1.5	−22.4 ± 1.9	−20.7 ± 1.7	−22.8 ± 0.5	−21.8 ± 0.3	−21.4 ± 0.5	−20.9 ± 0.5	−20.6 ± 0.3		
δ^34^S	17.7 ± 1.8	17.0 ± 2.0	19.9 ± 1.5	16.4 ± 0.6	13.9 ± 1.7	11.7 ± 0.3	10.7 ± 0.2	9.8 ± 0.7		
*Cyanea*										
δ^15^N							15.9 ± 3.0	12.4 ± 0.5	12.5 ± 0.5	15.3 ± 3.2
δ^13^C							−22.0 ± 0.6	−20.9 ± 0.2	−20.9 ± 0.5	−20.6 ± 0.4
δ^34^S							17.8 ± 0.3	18.4 ± 0.9	17.7 ± 0.8	18.4 ± 0.4

Maximum δ^15^N values of *A. aurita* were measured in June with 14.8 ± 2.3 ‰. These values then rapidly decreased to 10.9 ± 2.3 ‰ in early July (GAM, *F* = 15.4, *p* < 0.01), followed by a slight increase until the end of the period of occurrence in September (11.8 ± 0.7 ‰) (Fig. 2b). δ^15^N values of seston and zooplankton changed little over the observation period (GAM, *F* = 1.8, *p* = 0.5; *F* = 1.2, *p* = 0.3, respectively), ranging from 6.3 ± 1.3 ‰ and 6.6 ± 1.1 ‰ in early June to 4.0 ± 1.0 ‰ and 6.1 ± 1.1 ‰ in late July and 5.0 ± 0.9 ‰ and 6.5 ± 1.3 ‰ in late September, respectively (Table 2).

**Fig. 3:**
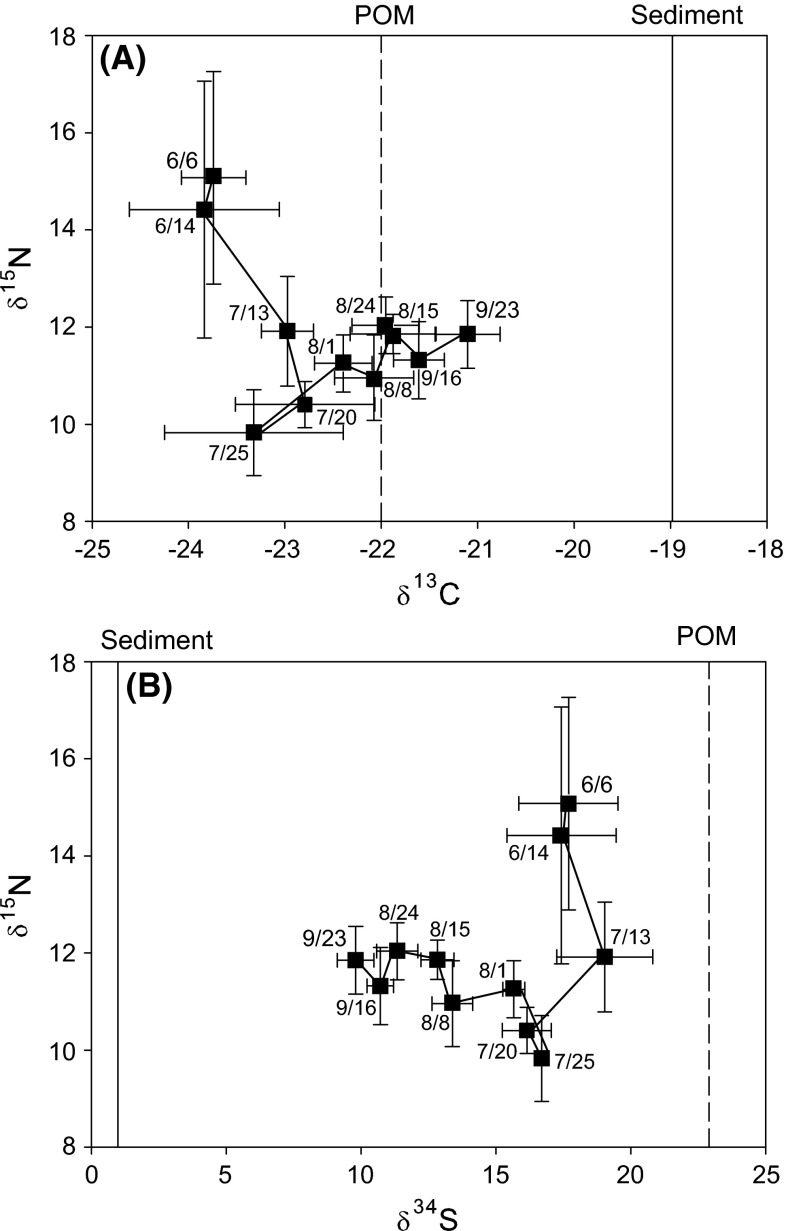
Stable isotope values of δ^13^C versus δ^15^N (**a**) and δ^34^S versus δ^15^N (**b**) for *A. aurita*. *Numbers* indicate the date of sampling. The *symbols* were connected in the temporal order of the data points, thus providing a time trajectory of change in isotope values

Temporal variation in δ^34^S of *A. aurita* was particularly pronounced, with a high in June and July (on average 17.4 ± 1.8 ‰ and 17.6 ± 2.0 ‰ respectively), followed by a steady decline (GAM, *F* = 45.3, *p* < 0.01) of more than 7 ‰ until late September (9.8 ± 0.7 ‰) (Fig. 2c). In contrast, δ^34^S of zooplankton decreased from 20.9 ± 1.0 ‰ in early June to 18.2 ± 1.1 ‰ in early July, followed by a slight increase to 19.1 ± 2.4 ‰ in September (GAM, *F* = 29.8, *p* > 0.01). Seston changed from 11.9 ± 3.2 in June to 12.4 ± 0.9 in late September. Temporal variation was significant (GAM, *F* = 5.2, *p* < 0.01), but of much lower magnitude than for *A. aurita*. To better illustrate temporal changes in SI composition of *A. aurita*, biplots of δ^13^C–δ^15^N and δ^15^N–δ^34^S with respect to sampling date are provided in Fig. 3.

**Fig. 4:**
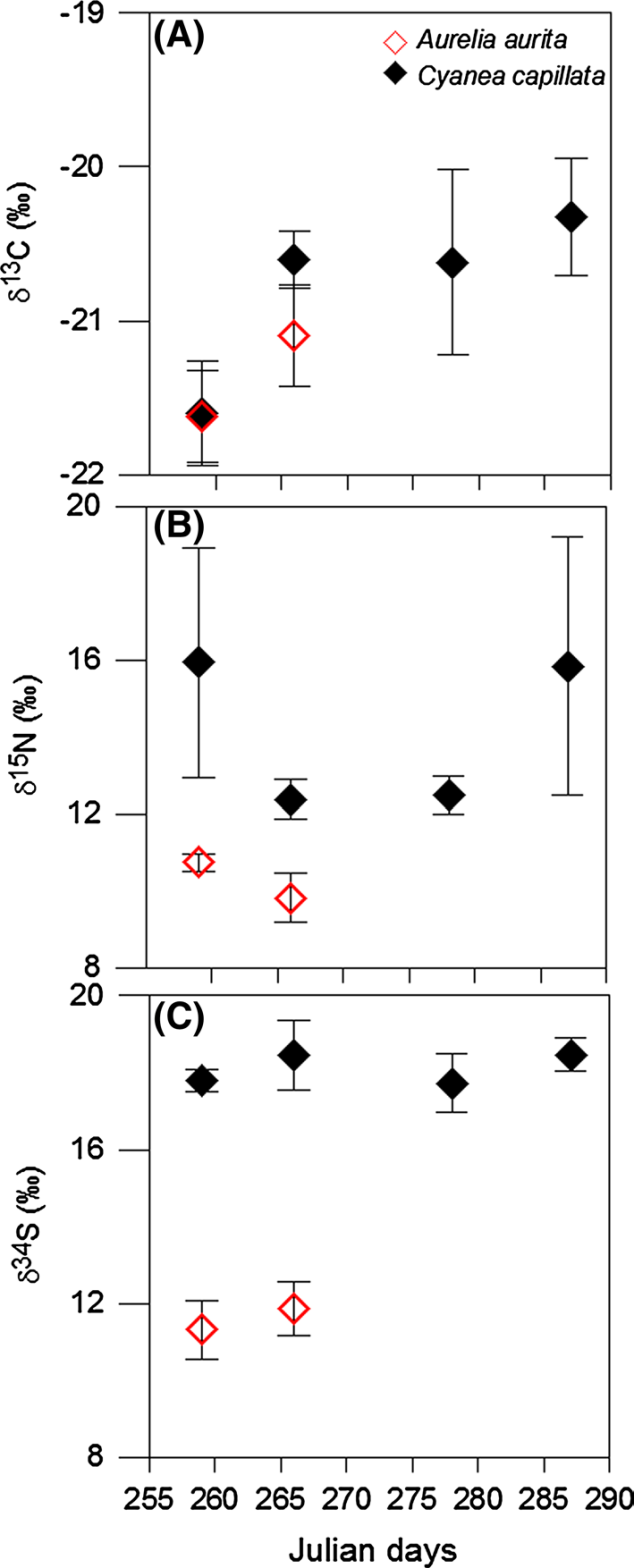
δ^13^C, δ^15^N and δ^34^S of *C. capillata* (*black diamond*) and *A. aurita* (*red diamond*) over the course of 2 months in the Kiel Fjord (Sep–Oct 2011). Julian day 255 corresponds to September 12 and 290 to October 17

In contrast to *A. aurita,C. capillata* showed fewer changes over the period of its occurrence in Kiel Fjord (Table 2; Fig. 4). There was a significant increase (ANOVA, *F*_(1,18)_ = 6.9, *p* = 0.01) in δ^13^C of *C. capillata* from September (−21.1 ± 0.6 ‰) to October (−20.5 ± 0.5 ‰), but no significant changes in δ^15^N or δ^*34*^*S* were measured. During the short period of species co-occurrence in September, the mean δ^13^C values of *C. capillata* were not significantly different from *A. aurita* (*F*_(1,18)_ = 1.6, *p* = 0.2), but δ^15^N (*F*_(1,17)_ = 8.1, *p* = 0.01) and δ^34^S (*F*_(1,18)_ = 632.7, *p* < 0.001) showed highly significant differences, and no evidence for an approximation of values over time.

### Contribution of prey sources to the diets of jellyfish

**Fig. 5:**
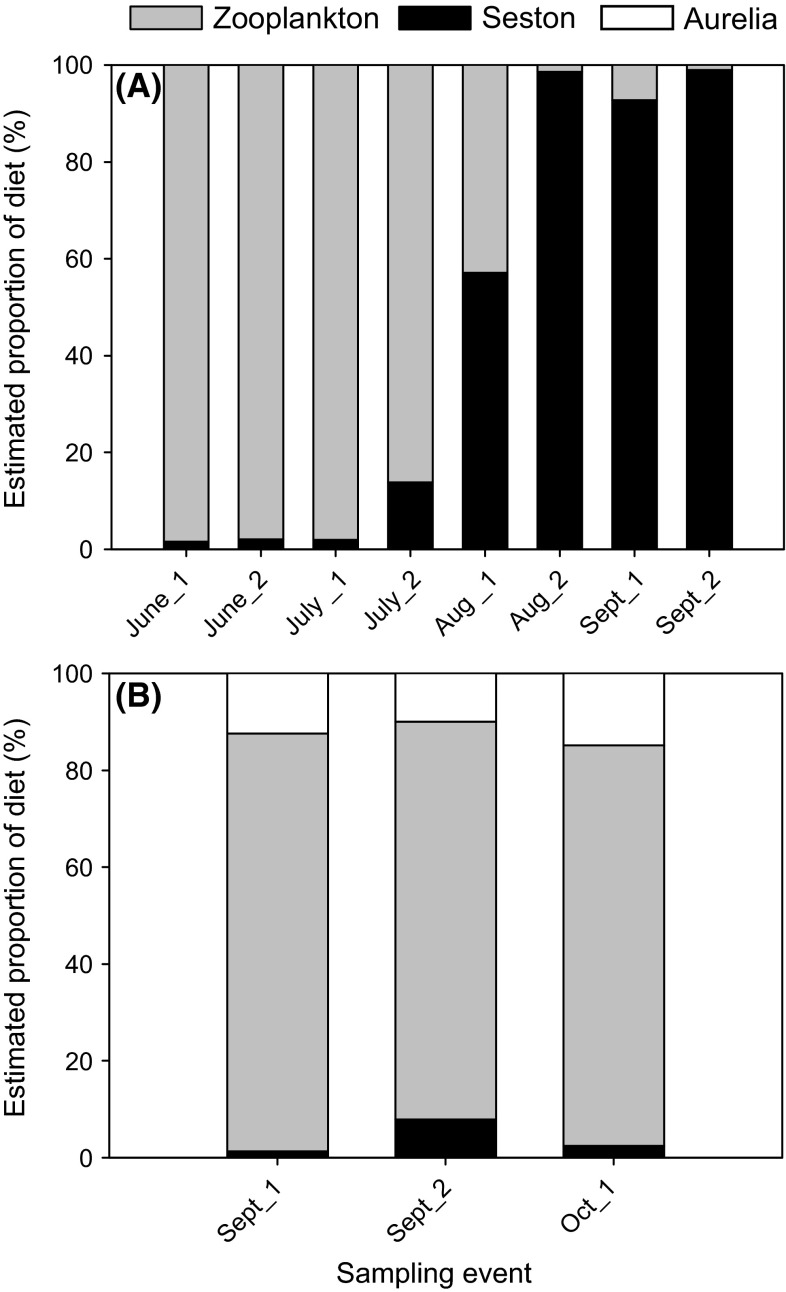
Graphical outcome of MixSIR models indicating percentage of mesozooplankton (*gray bar*) and microplankton (*black bar*) to the diet of *A. aurita* (**a**) and *C. capillata* (**b**) from June to October 2011 in a biweekly time span

Regarding the analysis of potential contributions of different prey sources to the dietary mix of *A. aurita*, and assuming that all potential food sources were included, the MixSIR mixing models indicated a drastic shift from a mesozooplankton based diet (96.6 ± 0.8 % of total possible food sources) to a seston based diet (99.8 ± 0.2 %) at the end of the growing season in September (Fig. 5a).

In contrast, the MixSIR mixing model for *C. capillata* indicated that this species fed mainly on mesozooplankton prey items over the limited period of observation in Kiel Fjord, whereas *A. aurita* comprised <15 % of its diet, and seston was nearly absent from its diet (Fig. 5b).

## Discussion

The pronounced shifts of ~3 ‰ in δ^13^C, ~4 ‰ in δ^15^N and the sharp decline of ~7 ‰ in δ^34^S within the same population of the jellyfish species *A. aurita* over a period of 4 months highlighted the potential for substantial intraspecific isotopic seasonal variation in jellyfish populations in their natural environment. This underscores the importance to account for such changes in SI feeding ecology studies on this group to avoid misinterpretation of datasets. Because the temporal changes of the SI values of potential prey items, in particular for δ^34^S, were much lower, it seems most likely that the dietary composition of *A. aurita* changed significantly over time. This interpretation was strengthened by the shift in *A. aurita* δ^13^C values which again differed from the pattern of the shifts in SI values of the potential prey.

δ^34^S of POM in Kiel Fjord was recorded at ~21 ‰, whereas sediment δ^34^S was at ~1 ‰ (Hansen et al. 2009). These two extremes represent the isotopic endpoints of potential food sources at the base of the local food webs, i.e., δ^34^S isotopic values of all components in Kiel Fjord food webs generally fall within this range. δ^34^S has therefore been used in previous studies as indicator of benthic versus pelagic dietary sources (see e.g., Jaschinski et al. 2011; Mittermayr et al. 2014a). While δ^34^S fractionation rates of jellyfish has not been reported so far, the strong shift to lower δ^34^S values of *A. aurita* over time may suggest a dietary shift from strictly pelagic to benthic food sources. We were unable to analyze gut contents to support this hypothesis; however, our mixing model results would be consistent with a switch from pelagic mesozooplankton as the main carbon source to benthic microplankton (e.g., protozoan) and/or resuspended organic particles from the benthos over the course of 4 months. In this context, considering the brief (2 week) duration and the weak nature of stratification during the study period, hydrographical changes probably were not a driver of the observed changes in SI values.

The changes observed in *A. aurita* SI values during its growing season in Kiel Fjord have important implications. Firstly, there is an ongoing debate in the field of isotope ecology regarding the need to account for species-specific temporal variation in isotopic values (Fleming et al. 2015; Jennings et al. 2008). Our finding confirm recent results by Fleming et al. (2015) with respect to substantial temporal variation in C and N values of jellyfish, and in addition highlighted particularly strong variation in S SI values over time that has not been previously assessed. The pronounced and rapid temporal changes observed here strongly underscore that SI feeding ecology studies in particular of jellyfish that do not account for this variation can result in misinterpretation of datasets. This point is illustrated by the fact that conclusions regarding the feeding ecology of *A. aurita* would be diametrically opposite when choosing only one isolated sampling point in June versus a point in September. Secondly, bentho-pelagic coupling is a key ecosystem process (Marcus 1998). Our data and the resulting mixing model suggest that in contrast to the exclusively planktonic feeding ecology commonly assumed for this species (Behrends and Schneider 1995; Hansson et al. 2005; Moller and Riisgard 2007), it may also depend on benthic food sources at the base of its food web (see also Pitt et al. 2008). This would have consequences for assessments of the ecological role and impact of jellyfish and should be considered in the parameterization of food web models.

While the period of overlap of *A. aurita* with *C. capillata* was relatively short, this study nevertheless provides the first insights regarding the trophic interactions between these two species in Kiel Fjord based on SI analysis. Previously, based on both field and experimental observations, *C. capillata* has mainly been described as an important predator of *A. aurita* (Bamstedt et al. 1994; Hansson 1997; Titelman et al. 2007), although Hansson concluded that assimilation rate estimates were needed to clearly define the proportion of *A. aurita* in the diet. In contrast, while the short temporal overlap and the absence of significant growth of *C. capillata* means that this result needs to be treated with caution, our data provide an indication that the role of *A. aurita* in *C. capillata* diet may be lower than previously thought. At the time of first occurrence in Kiel Fjord in September, the δ^34^S values of *C. capillata* were significantly higher (+ ~8 ‰) than the values of *A. aurita*. Over the following period of overlap, no temporal approximation in δ^34^S values—which would be expected under the scenario of *C. capillata* feeding on *A. aurita* and assuming that turnover rates reported by D’Ambra et al. (2014) for *A. aurita* do apply—occurred. Instead, *C. capillata* δ^34^S isotope values remained close to pelagic isotopic ratios, which were reflected by the estimated contribution of *A. aurita* to the diet of *C. capillata* of only 15 % as indicated by the MixSIR model.

To conclude, this study demonstrates the potential of triple stable isotope datasets to gain novel insights into the feeding ecology and ecological role of jellyfish, which is urgently needed due to the rising concern about worldwide increases in this marine ecosystem component in the course of global change (Gibbons and Richardson 2013). Furthermore, carefully designed experimental designs are required in order to account for potential temporal variation in consumers and their prey to unlock the full potential future of such approaches.

## Limitations of the study

The data reported here support the assumption that diet composition of *A. aurita* has changed over time not only from mesozooplankton to microzooplankton food, but also from a more pelagic source to a benthic one. It is important to mention that the MixSIR model results leading to this conclusion were mainly driven by the significant change in δ^34^S values of *A. aurita*. Results in δ^13^C and δ^15^N do not contradict this conclusion, but taken by themselves would have allowed different interpretations as well. In particular, the offset between *A. aurita* and the two assumed dietary source categories (zooplankton and seston) is always larger than >5 ‰. We assume here that this difference is due to trophic fractionation, which would place *A. aurita* on the upper end of the range reported for other organisms and larger than the value previously reported by D’Ambra et al. (2014). An alternative explanation would be the presence of an additional trophic complexity, e.g., an unidentified dietary source with a higher δ^15^N value and similar δ^34^S value compared to seston not included in our mixing model, although the low δ^34^S values would then still support a more benthic origin of material at the base of the food web in fall (Jaschinski et al. 2008; Mittermayr et al. 2014b).

Regarding our conclusion of limited feeding of *C. capillata* on *A. aurita*, it is important to consider that fractionation rates in particular for δ^34^S, and for jellyfish feeding on other gelatinous prey, have not been reported, i.e., we are assuming that general relationships in isotope ecology will hold true; however, this assumption needs validation in the future. Again, δ^13^C and δ^15^N values do not contradict this result, but based on C and N alone, a higher importance of *A. aurita* in the prey would have been a possible solution as well.

Finally, as in other isotope ecology studies, it is important to consider that SI fractionation factors may in part depend on the physiological state and the sexual maturity of an organism. However, while the effect of metabolic change on turnover rates has been assessed (Bearhop et al. 2004), little information exists on changes in fractionation. For practical reasons, rates are therefore commonly assumed as stable over time in SI feeding ecology studies (e.g., Michener and Kaufman 2007). *A. aurita* developed gonads in mid-June and its sexual reproduction started in late July (unpublished data) which likely explain the observed slight C:N decrease of *A. aurita* during this time (Milisenda et al. 2014). It is still unclear to which extent the reproductive stage of jellyfish might influence SI fractionation factors, but as no obvious pattern coincided with the timing of reproduction here, we considered the effects as limited.

## Electronic supplementary material

Below is the link to the electronic supplementary material.

Supplementary material 1 (PDF 153 kb)
