# Supplementary material for: Temporal dietary shift in jellyfish revealed by stable isotope analysis
Source: Mar Biol. 2016 Apr 22;163:112. doi: 10.1007/s00227-016-2892-0 (PMC4841851; doi:10.1007/s00227-016-2892-0)

Electronic Supplementary Material for Marine Biology research article

Temporal dietary shift in jellyfish revealed by stable isotope analysis

Jamileh Javidpour[1]*, Ashlie N. Cipriano-Maack[2], Agnes Mittermayr[3], Jan Dierking[1]

* Corresponding author: jjavid@geomar.de

[1] GEOMAR Helmholtz Centre for Ocean Research Kiel, Düsternbrooker Weg 20, 24105 Kiel, Germany

[2] University College Cork, Biological, Earth and Environmental Science, Cooperage Building, Distillery Fields, North Mall, Cork, Ireland

[3] Marine Biological Laboratory, 7 MBL Street, Woods Hole, MA 02543, USA

S. 1: : δ^{13}C correction for lipids using two methods of D'Ambra et al. 2014 (diamonds) and Post et al. (triangles) compared to our raw data of this study (cross signs).

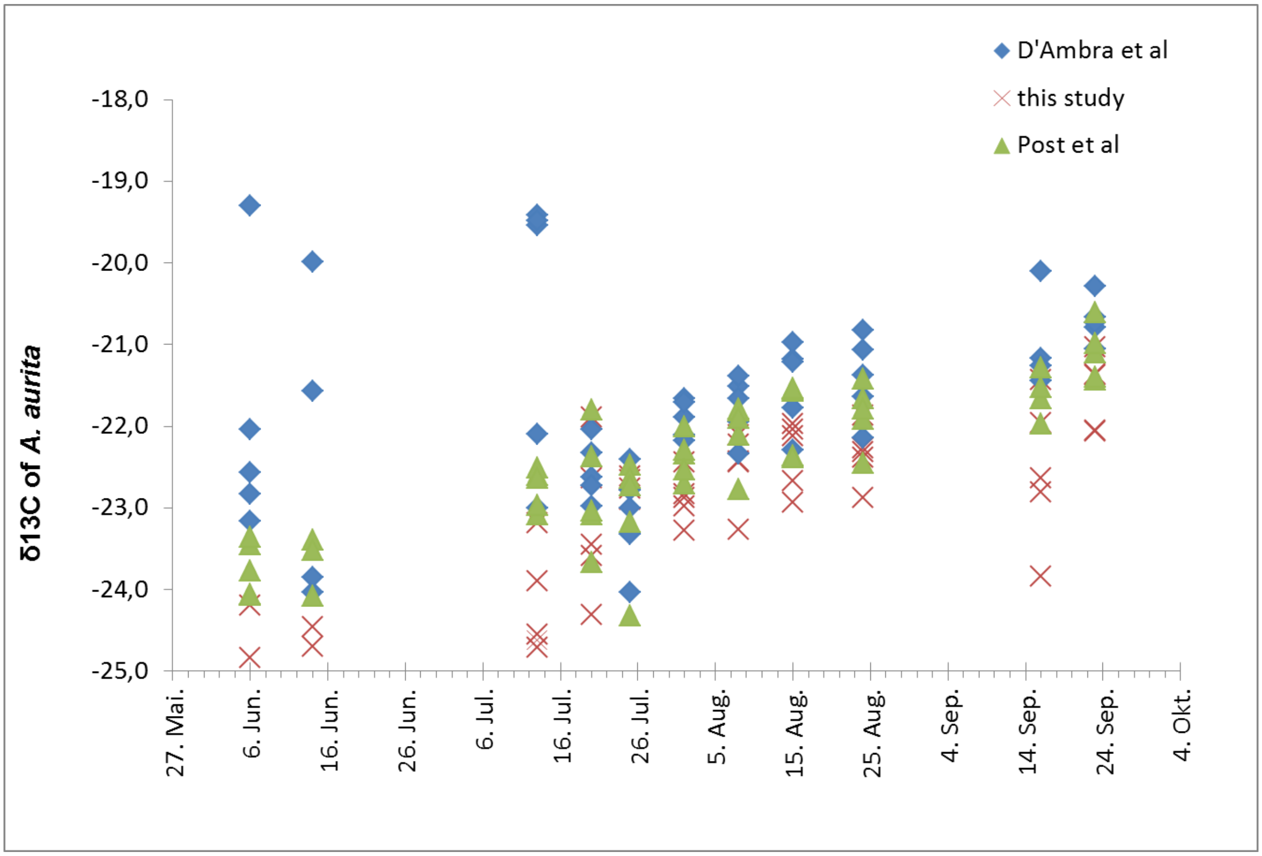

Supplement: Supplementary file 1 — Supplementary material 1 (PDF 153 kb) [file 227_2016_2892_MOESM1_ESM.pdf]
